# Relative Contributions of Functional Capacity and Inflammatory Activity to Quality of Life in Heart Failure with Preserved Ejection Fraction

**DOI:** 10.3390/biomedicines14061270

**Published:** 2026-06-02

**Authors:** Vladimir Zdravković, Đorđe Stevanović, Goran Davidović, Ivan Simić, Marijana Stanojević-Pirković, Željko Ivošević, Nina Uraković, Lidija Stojanović, Isidora Stanković, Neda Ćićarić, Sara Milojević, Mladen Maksić, Katarina Radojević, Marija Popović

**Affiliations:** 1 Cardiology Clinic, University Clinical Center Kragujevac, 34000 Kragujevac, Serbia; vladazdrav@gmail.com (V.Z.); medicusbg@yahoo.com (G.D.); ivansimickg@gmail.com (I.S.); n.urakovic@gmail.com (N.U.); likistojanovic@gmail.com (L.S.); isidora.st.md@gmail.com (I.S.); nedaa.cicaric@gmail.com (N.Ć.); marijapopovickg@gmail.com (M.P.); 2 Department of Internal Medicine, Faculty of Medical Sciences, University of Kragujevac, 34000 Kragujevac, Serbia; zeljkoivosevic274@gmail.com (Ž.I.); asussonicmaster95@gmail.com (M.M.); 3 Center for Laboratory Diagnostics, University Clinical Center Kragujevac, 34000 Kragujevac, Serbia; marijanas14@gmail.com; 4 Department of Medical Biochemistry, Faculty of Medical Sciences, University of Kragujevac, 34000 Kragujevac, Serbia; 5 Clinic for Endocrinology, Diabetes and Metabolic Disease, University Clinical Centre Kragujevac, 34000 Kragujevac, Serbia; 6 Department of Pharmacology and Toxicology, Faculty of Medical Sciences, University of Kragujevac, 34000 Kragujevac, Serbia; 7 Department of Pharmacology, Clinical Pharmacology and Toxicology, Faculty of Medicine, University of Belgrade, 11000 Belgrade, Serbia; saramiloesp@gmail.com; 8 Clinic for Gastroenterology and Hepatology, University Clinical Centre Kragujevac, 34000 Kragujevac, Serbia; 9 Pulmonology Clinic, University Clinical Center Kragujevac, 34000 Kragujevac, Serbia; radojevickatarina3@gmail.com

**Keywords:** functional capacity, inflammation, interleukin-6, heart failure with preserved ejection fraction, quality of life

## Abstract

**Background/Objectives**: Impaired quality of life (QoL) represents one of the most important clinical determinants in heart failure with preserved ejection fraction (HFpEF). This study aimed to evaluate the incremental explanatory value of functional performance and inflammatory biomarkers for QoL in a clinically stable HFpEF cohort. **Methods**: A single-center observational study enrolled 110 consecutive patients with stable HFpEF. Functional capacity was assessed using the six-minute walk test (6MWT), expressed mainly as percentage of predicted distance. Health-related QoL was measured using the EQ-5D-5L utility index (primary outcome). Circulating IL-6, CRP, and NT-proBNP were obtained from peripheral blood. Hierarchical multivariable linear regression was applied to quantify the incremental contribution of clinical variables, functional capacity, and biomarkers. **Results**: The median age was 72 years, and 52.7% of the participants were women. The median 6MWT distance was 340 m (75.9% of predicted), and the median EQ-5D index was 0.76. The baseline clinical regression model (age, sex, atrial fibrillation, and glomerular filtration rate) explained 23.5% of EQ-5D variance. The addition of functional capacity increased explained variance to 45.2% (ΔR^2^ = +0.217). The inclusion of IL-6 and NT-proBNP provided a modest additional increase (R^2^ = 0.468; ΔR^2^ = +0.042 in addition to Model 2). In the fully adjusted model, functional capacity (β = 0.376, *p* < 0.001) and IL-6 (β = −0.185, *p* < 0.05) remained independent predictors, whereas NT-proBNP lost significance. **Conclusions**: In stable HFpEF, objective functional capacity represents the dominant determinant of QoL, while inflammatory activation provides an independent but smaller contribution. Functional assessment may therefore be central to patient-centered phenotyping and therapeutic targeting.

## 1. Introduction

Heart failure with preserved ejection fraction (HFpEF) now accounts for nearly half of the global heart failure population, and its prevalence continues to rise in parallel with population ageing and the growing burden of cardiometabolic comorbidities. Despite preserved left ventricular ejection fraction, patients frequently experience substantial symptom burden, impaired exercise tolerance and reduced quality of life (QoL), while disease-modifying therapies remain limited and management is largely focused on symptom control and functional stabilization [1,2,3,4].

Health-related QoL has therefore emerged as a clinically meaningful endpoint in HFpEF, reflecting not only symptom severity but also functional autonomy and daily activity limitations. Observational studies and clinical trials have demonstrated that QoL impairment in HFpEF is comparable to that observed in heart failure with reduced ejection fraction (HFrEF) and is independently associated with hospitalization and mortality [5,6,7,8,9,10]. However, conventional clinical markers, including New York Heart Association (NYHA) functional class and natriuretic peptides, only partially capture the heterogeneity of disease mechanisms and show limited sensitivity for explaining inter-individual variability in patient-reported outcomes [1,11,12].

Impaired exercise tolerance constitutes a central clinical phenotype of HFpEF and a principal driver of symptom perception and functional restriction. Its pathophysiology is multifactorial, encompassing impaired diastolic reserve, ventricular–arterial uncoupling, chronotropic incompetence, coronary microvascular dysfunction and peripheral skeletal muscle abnormalities [13,14,15,16]. Objective assessment using the six-minute walk test (6MWT) provides an integrated measure of cardiopulmonary and peripheral functional reserve and correlates with functional capacity and prognosis [15,17,18]. Functional performance therefore constitutes a plausible mechanistic link between hemodynamic impairment and perceived quality of life.

Concurrently, HFpEF is increasingly conceptualized as a systemic inflammatory syndrome driven by comorbidity-related endothelial and microvascular dysfunction. Chronic low-grade inflammation promotes myocardial stiffness, interstitial fibrosis and impaired nitric oxide signaling, while also influencing skeletal muscle metabolism and peripheral oxygen utilization [19,20,21,22,23,24]. Circulating inflammatory biomarkers, including C-reactive protein (CRP) and interleukin-6 (IL-6), have been associated with symptom severity, reduced exercise capacity and adverse outcomes in HFpEF [19,21,24,25,26,27,28,29,30,31,32]. Nevertheless, the incremental contribution of inflammatory activation to QoL impairment beyond objective functional limitation remains insufficiently characterized, particularly in real-world cohorts.

Most prior studies have examined functional capacity, biomarkers and QoL in isolation or within selected trial populations, often with limited multivariable adjustment and heterogeneous measurement instruments [5,12]. Integrated analyses quantifying the relative explanatory value of clinical factors, functional performance and biological activity for patient-reported outcomes are scarce.

Accordingly, the present study aimed to investigate the determinants of health-related QoL in a clinically stable HFpEF cohort, with a particular focus on the interplay between objective functional capacity and inflammatory activity. Using hierarchical multivariable modeling, we sought to quantify the incremental contribution of functional performance beyond baseline clinical characteristics and to assess whether inflammatory biomarkers provide additional independent explanatory value for QoL impairment.

## 2. Materials and Methods

### 2.1. Study Design and Population

This study was designed as a clinical, non-interventional, observational cohort study. The study population consisted of adult patients with a confirmed diagnosis of HFpEF treated at the Department of Cardiology, University Clinical Center Kragujevac (Kragujevac, Serbia). All the study procedures were conducted in accordance with the principles of Good Clinical Practice and applicable national and international ethical regulations. The study protocol was approved by the institutional ethics committee of the University Clinical Center Kragujevac. All the participants provided written informed consent prior to enrollment.

HFpEF was diagnosed according to the contemporary European Society of Cardiology guidelines, based on the following criteria [33]:

- presence of signs (e.g., elevated jugular venous pressure, pulmonary crackles, and peripheral edema) and/or symptoms (e.g., breathlessness, ankle swelling, and fatigue) of heart failure;
- left ventricular ejection fraction ≥ 50%; and
- objective evidence of structural and/or functional cardiac abnormalities consistent with left ventricular diastolic dysfunction or elevated filling pressures, including elevated natriuretic peptide levels.

Patients were consecutively recruited over a ten-month period (November 2023 to August 2024), in accordance with predefined inclusion and exclusion criteria.

Inclusion and Exclusion Criteria

Inclusion criteria:

- Age ≥ 18 years;
- Confirmed diagnosis of HFpEF;
- Clinically stable disease, defined as at least four weeks without:
  (i) hospitalization for heart failure decompensation;
  (ii) urgent specialist evaluation requiring treatment escalation for heart failure;
  (iii) unscheduled outpatient visit due to worsening heart failure symptoms;
  (iv) self-initiated increase in diuretic dose.

Exclusion criteria:

- Refusal to participate;
- Modification of cardiovascular therapy within four weeks prior to assessment;
- Absence of guideline-directed medical therapy (GDMT) during the four weeks preceding evaluation, mostly implying sodium–glucose cotransporter-2 inhibitors and symptomatic diuretic therapy if needed;
- Acute or recent infection within four weeks;
- Acute coronary syndrome within three months;
- Cerebrovascular event within three months;
- Systemic connective tissue disease;
- Neurological or psychiatric disorders;
- Inflammatory bowel disease;
- Infiltrative cardiomyopathies (e.g., sarcoidosis, amyloidosis), primary hypertrophic cardiomyopathy, arrhythmogenic right ventricular cardiomyopathy, Takotsubo cardiomyopathy, or active myocarditis;
- Severe pulmonary dysfunction (forced vital capacity < 60% of predicted within the preceding year);
- Severe anemia (hemoglobin < 80 g/L);
- Terminal malignancy;
- End-stage renal disease (estimated creatinine clearance < 15 mL/min/1.73 m^2^);
- Severe aortic and/or mitral valve stenosis or regurgitation.

### 2.2. Variables

#### 2.2.1. Clinical and Laboratory Assessment

- Sociodemographic characteristics and comorbidities were obtained through structured interviews and review of medical records.
- Laboratory analyses were performed according to institutional standards and included complete blood count, coagulation parameters, routine biochemical profile, inflammatory markers, and cardiac biomarkers. In the present analysis, selected biomarkers with established pathophysiological relevance to HFpEF and quality of life were analyzed and reported: CRP, IL-6, and NT-proBNP. Renal function was assessed using estimated glomerular filtration rate (eGFR).
- Clinical assessment included physical examination with quantification of pretibial edema using the pitting scale, assessment of symptom duration and frequency. Functional classification was performed according to the New York Heart Association (NYHA), as follows: (I) No limitation of physical activity. Ordinary physical activity does not cause undue breathlessness, fatigue, or palpitations. (II) Slight limitation of physical activity. Comfortable at rest, but ordinary physical activity results in undue breathlessness, fatigue, or palpitations. (III) Marked limitation of physical activity. Comfortable at rest, but less than ordinary activity results undue breathlessness, fatigue, or palpitations. (IV) Unable to carry on any physical activity without discomfort. Symptoms at rest can be present. If any physical activity is undertaken, discomfort is increased.

#### 2.2.2. Assessment of Functional Capacity

Exercise tolerance was evaluated using the standardized 6MWT. For each participant, the predicted reference walking distance was calculated based on sex, age, height, and body weight using validated equations, according to the American Thoracic Society (ATS) statement: (a) for males: (7.57 × body height [cm]) − (5.02 × age [years]) − (1.76 × body weight [kg]) − 309; (b) for females: (2.11 × body height [cm]) − (2.29 × body weight [kg]) − (5.78 × age [years]) + 667) [34]. Functional capacity was expressed as both absolute walking distance and percentage of predicted distance. Perceived exertional dyspnea during the test was quantified using the Borg scale [35].

#### 2.2.3. Assessment of Health-Related Quality of Life

Health-related quality of life was assessed using the EQ-5D-5L instrument, for which formal permission was obtained from the copyright holder. The descriptive system evaluates five dimensions (mobility, self-care, usual activities, pain/discomfort, and anxiety/depression), each graded on a five-level Likert scale. The EQ visual analogue scale (EQ-VAS) reflects the participant’s self-rated overall health state on a 0–100 scale. The EQ-5D-5L utility index was calculated using the England (United Kingdom) EQ-5D-5L value set, yielding a continuous index score ranging from health states death (0) to full health (1) [36]. The EQ-5D-5L utility index, treated as a continuous variable, was observed as the primary outcome of the study.

### 2.3. Statistical Analysis

Statistical analyses were performed using SPSS software (version 25.0, IBM Corp., Armonk, NY, USA). Normality of continuous variables was assessed using the Kolmogorov–Smirnov test. Categorical variables are presented as absolute and relative frequencies. Continuous variables are presented as mean ± standard deviation for normally distributed data or median with interquartile range for non-normally distributed data. Data were summarized using tabular and graphical methods. Between-group comparisons for categorical variables were performed using the chi-square test or Fisher’s exact test, as appropriate. Comparisons of continuous variables were conducted using the independent-samples t-test or Mann–Whitney U test, depending on data distribution. Correlations between continuous variables were assessed using Spearman’s correlation coefficients. Biomarkers exhibiting skewed distributions were log-transformed prior to regression analyses.

### 2.4. Multivariable Modeling Strategy

The primary outcome was the EQ-5D utility index treated as a continuous variable. Associations between candidate predictors and quality of life were first explored using univariable analyses. Intercorrelations among predictors were examined to assess potential multicollinearity; highly correlated variables (absolute correlation coefficient ≥ 0.70 or variance inflation factor > 5) were not simultaneously included in multivariable models. Objective functional capacity was prioritized over subjective symptom measures to minimize redundancy. Selection of continuous variables was based on strength of association with the outcome and contribution to model performance. Candidate variables, including key HFpEF-related comorbidities, were initially evaluated in univariable analyses; however, selection for multivariable modeling was not based solely on statistical significance. Variables were retained based on a combination of clinical relevance, avoidance of overfitting given the sample size, and their contribution to overall model performance. To minimize redundancy and multicollinearity, closely related clinical variables were not simultaneously included in the final models. Given the sample size, the number of predictors included in the final model was intentionally limited to ensure model stability.

Candidate predictors were first evaluated in univariable analyses. However, univariable statistical significance was not used as the sole criterion for inclusion or exclusion from multivariable models. Given the pathophysiological relevance of cardiometabolic comorbidities in HFpEF, variables such as obesity, diabetes mellitus, hypertension, atrial fibrillation, renal dysfunction, and global comorbidity burden were clinically considered during model construction. Because of the moderate sample size and the risk of overfitting, the number of predictors in the final hierarchical models was intentionally limited. Renal dysfunction was represented by continuous eGFR; atrial fibrillation was retained as a clinically relevant covariate; comorbidity burden and individual cardiometabolic comorbidities were evaluated in the sensitivity analyses, which were performed by individually adding key cardiometabolic comorbidities and/or global comorbidity burden to the fully adjusted model (Supplementary Table S1).

Hierarchical multivariable linear regression was applied to evaluate the incremental contribution of clinically meaningful domains. In the first block, demographic and clinical covariates were entered to establish a baseline clinical model. In the second block, objective functional capacity was added. In the third block, inflammatory and cardiac biomarkers were introduced. Continuous variables were computed using the ln(x) function if needed. Model performance was evaluated using adjusted R^2^ and change in R^2^ between blocks. Multicollinearity was assessed using variance inflation factors. Model assumptions were evaluated through residual diagnostics, including normality, homoscedasticity, and influence statistics. All statistical tests were two-sided, and *p*-values < 0.05 were considered statistically significant. Given the number of predictors included in the final model (k = 6) and the sample size (n = 110), the study satisfied commonly recommended sample size criteria for multiple linear regression analysis. Specifically, the ratio of observations to predictors (n:k) exceeded 15:1, and the sample size met Green’s rule (n ≥ 50 + 8k), supporting adequate model stability [37].

## 3. Results

### 3.1. Baseline Characteristics

A total of 110 patients with clinically stable HFpEF were included in the analysis. The cohort was predominantly elderly, with a median age of 72.0 years (IQR 68.0–76.0), with a slight female predominance (52.7%). Cardiovascular comorbidity burden was substantial: arterial hypertension was present in 94.5%, hyperlipidemia in 72.7%, diabetes mellitus in 45.5%, and atrial fibrillation in 41.8% of patients. Chronic kidney disease, defined as eGFR < 60 mL/min/1.73 m^2^, was observed in 37.3%. More than half of the cohort was obese according to BMI, with a median Charlson Comorbidity Index of 4.0 (IQR 3.0–5.0). The median duration of heart failure symptoms prior to study inclusion was 15.0 months. In addition, the median time from symptom onset to definitive diagnosis was 12.0 months, while 45.5% of patients were diagnosed during hospitalization for heart failure decompensation. Most patients were classified as NYHA functional class II (73.6%), and peripheral edema was present in 80.0% of participants (70/110 in the pitting 1 and 18/110 patients in the pitting 2 scale). Inflammatory and cardiac biomarkers demonstrated moderate elevation, with median CRP 5.1 mg/L (IQR 1.7–6.8), IL-6 5.4 pg/mL (IQR 3.0–7.7), and NT-proBNP 566 pg/mL (IQR 272–1370). The median eGFR was 65.5 mL/min/1.73 m^2^ (IQR 49.5–82.2) (Table 1).

### 3.2. Functional Capacity and Quality of Life

Functional performance assessed by the 6MWT demonstrated a median walking distance of 340 m (IQR 280–392), corresponding to 75.9% (IQR 62.7–83.8) of the predicted distance. Perceived exertion at the cohort level was moderate, with a median Borg score of 3.5 (IQR 3.0–5.0), while 34.5% of patients reported severe dyspnea, defined as Borg ≥ 5. Health-related QoL was moderately impaired. The median EQ-5D index was 0.76 (IQR 0.67–0.88), while the EQ-5D-5L VAS score was 60.0% (IQR 50.0–61.2). Only two patients (1.8%) reported no problems across all EQ-5D dimensions. Within the mobility domain, only 15.5% of patients reported no limitations, whereas 34.5% reported moderate and 6.4% severe mobility problems. The results are presented in the Table 2. Data regarding other EQ-5D-5L domains are provided in Supplementary Table S1.

### 3.3. Univariable Associations with Quality of Life

In univariable analyses, lower EQ-5D index values were associated with female sex (median 0.73 vs. 0.80 in males, *p* < 0.05), presence of atrial fibrillation (0.73 vs. 0.78, *p* < 0.05), higher NYHA functional class (NYHA III: 0.53 vs. NYHA II: 0.81, *p* < 0.001), and presence of peripheral edema (0.74 vs. 0.87, *p* < 0.05). Increasing age (ρ = −0.245, *p* < 0.05) and higher Charlson Comorbidity Index (CCI) (ρ = −0.308, *p* < 0.05) were also negatively correlated with quality of life. Objective functional capacity showed a strong positive association with EQ-5D index (percentage of predicted 6MWT distance covered: ρ = 0.496, *p* < 0.001), whereas perceived exertion was inversely related (Borg scale: ρ = −0.596, *p* < 0.001). Renal function correlated positively with quality of life (ρ = 0.357, *p* < 0.001). Inflammatory and cardiac biomarkers were consistently associated with worse quality of life, including CRP (ρ = −0.369, *p* < 0.001), IL-6 (ρ = −0.441, *p* < 0.001), and NT-proBNP (ρ = −0.426, *p* < 0.001). BMI ≥ 30 kg/m^2^ (*p* = 0.766), as well as other relevant comorbidities were not significantly associated with EQ-5D index in the univariable analysis (Table 3). However, decisions regarding multivariable model construction were not based solely on univariable *p*-values. Instead, variables were additionally selected according to clinical relevance, avoidance of redundancy, multicollinearity assessment, and model stability considerations. Key HFpEF-related comorbidities were further addressed in sensitivity analyses. 

### 3.4. Quality of Life Predictors

Hierarchical linear regression was performed to identify independent determinants of health-related quality of life. Variable selection was guided by clinical relevance, univariable associations, and assessment of multicollinearity. Highly correlated variables were not entered simultaneously. Objective functional capacity was prioritized over subjective symptom measures, and IL-6 was selected as the primary inflammatory biomarker. Hierarchical modeling was applied to evaluate the incremental contribution of clinical covariates, functional capacity, and biological markers to health-related quality of life. In Model 1, including demographic and clinical covariates (age, sex, atrial fibrillation, and eGFR), the model explained approximately 23.5% of the variance in EQ-5D index (R^2^ = 0.235). Female sex, atrial fibrillation, and higher eGFR remained independently associated with EQ-5D index, whereas age was not independently significant. In Model 2, the addition of objective functional capacity (percentage of predicted 6MWT distance covered) substantially improved model performance, increasing the explained variance to 45.2% (R^2^ = 0.452; ΔR^2^ = +0.217). Functional capacity emerged as the strongest independent predictor of QoL, while the association with atrial fibrillation was attenuated after adjustment for exercise performance. In Model 3, further inclusion of inflammatory and cardiac biomarkers (log-transformed IL-6 and NT-proBNP) led to a modest but statistically significant additional increase in explained variance (R^2^ = 0.468; ΔR^2^ = +0.042). IL-6 remained independently and inversely associated with EQ-5D index, whereas NT-proBNP did not retain independent significance after adjustment for functional capacity and inflammation. Functional capacity remained a robust independent determinant of QoL across all models. No relevant multicollinearity was detected in any of the models (Table 4). Sensitivity analyses were performed to evaluate whether the exclusion of selected cardiometabolic comorbidities influenced the primary findings. When obesity, diabetes mellitus, hypertension, and Charlson Comorbidity Index were individually added to the fully adjusted model, the associations of percentage of predicted 6MWT distance and IL-6 with EQ-5D index remained unchanged. None of these additional models significantly altered the overall interpretation of the primary hierarchical regression analysis. These results are presented in Supplementary Table S2.

**Figure 1:**
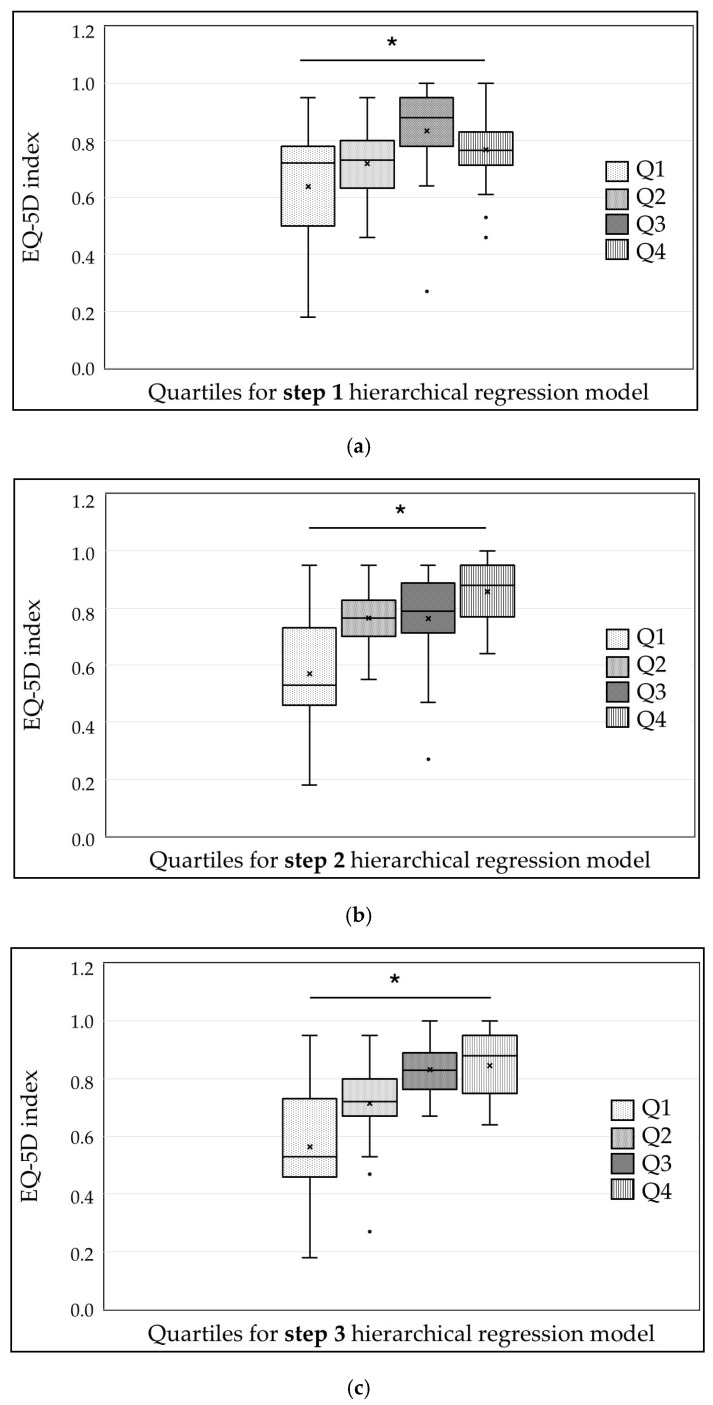
Distribution of observed EQ-5D index values across quartiles of predicted values derived from hierarchical regression models, showing a progressive increase in QoL scores observed across quartiles in Models 2 and 3, with “*” representing statistical significance at <0.05 level. Panels: (**a**) Step 1—demographics and comorbidities. (**b**) Step 2—addition of functional capacity. (**c**) Step 3—addition of cardiac and inflammatory biomarkers.

To further illustrate the discriminative performance of the regression models, predicted EQ-5D index values were categorized into quartiles and compared with the observed EQ-5D index using boxplot visualization (Figure 1). In the baseline clinical model (Model 1), separation between quartiles was modest and non-monotonic. Participants in the third quartile (50th–75th percentile of predicted values) exhibited the highest observed EQ-5D index, exceeding the values observed in the first (*p* = 0.030), second (*p* = 0.049), and fourth (*p* = 0.029) quartiles. The fourth quartile demonstrated significantly higher EQ-5D index values compared with the first (*p* < 0.001) and second (*p* = 0.001) quartiles, whereas no significant difference was observed between the first and second quartiles (*p* = 0.277). In contrast, both the functional model (Model 2) and the fully adjusted model (Model 3) demonstrated a graded increase in observed EQ-5D index across increasing prediction quartiles. Each successive quartile was associated with significantly higher observed quality-of-life values compared with the preceding quartile, indicating improved discriminative stratification and monotonic risk separation after inclusion of functional capacity and biological markers.

## 4. Discussion

The present study provides an integrated evaluation of clinical characteristics, objective functional capacity, and inflammatory activity as determinants of health-related QoL in a clinically stable HFpEF cohort. The principal finding is that objective functional performance, quantified by the percentage of predicted 6MWT distance, represents the dominant independent determinant of QoL, accounting for a substantial incremental proportion of explained variance beyond baseline demographic and clinical covariates. In contrast, inflammatory activation, represented by circulating IL-6, contributed a smaller but statistically independent explanatory component, whereas NT-proBNP did not retain independent significance after adjustment for functional performance and inflammation.

The novelty of the present study lies not in the use of functional capacity per se but in the integrative modeling approach that combines demographic factors, objective functional performance, and circulating biomarkers to evaluate their relative and incremental contributions to health-related QoL in HFpEF. While 6MWT has long been recognized as a marker of functional status, our findings demonstrate its dominant role over traditional clinical variables and its relationship with inflammatory and neurohormonal pathways in shaping patient-reported QoL. In particular, the hierarchical modeling strategy allowed differentiation between independent and non-independent associations, highlighting that certain biomarkers (e.g., NT-proBNP) lose apparent significance after accounting for functional capacity. These findings provide clinically relevant insight into the relative importance of functional limitation as a central determinant of QoL in HFpEF, supporting a patient-centered framework that prioritizes functional assessment alongside traditional clinical evaluation.

The clinical profile of the studied cohort reflects a typical contemporary HFpEF population characterized by advanced age, high cardiometabolic comorbidity burden, and a substantial prevalence of atrial fibrillation and chronic kidney disease, consistent with large registries [2,38,39,40]. Despite predominantly moderate symptom severity (73.6% in NYHA class II), patients exhibited marked heterogeneity in objective symptom burden, functional capacity and QoL impairment, underscoring the dissociation between perceived NYHA class and physiological limitation in HFpEF [5,11,12]. All the patients were clinically stable and receiving guideline-directed medical therapy, including loop diuretics, with no evidence of heart failure worsening or diuretic up-titration for at least four weeks prior to assessment, as defined by the inclusion criteria. Although peripheral edema was present in a high proportion of patients (80%), it was predominantly mild (pitting grade 1 in 63.6%), consistent with a clinically stable HFpEF population. Importantly, a prolonged delay between symptom onset and definitive diagnosis was observed in a substantial proportion of patients, with many individuals receiving the diagnosis only during hospitalization for decompensation. This finding highlights the persistent challenges in early recognition of HFpEF in routine clinical practice, driven by non-specific symptomatology, multimorbidity, and limited access to structured diagnostic algorithms [1,40,41,42,43,44]. Delayed diagnosis may contribute to prolonged symptom burden, functional decline, and impaired QoL prior to initiation of targeted management and comorbidity optimization [45].

Health-related QoL in the present study was assessed using the EQ-5D-5L instrument, which represents an important study limitation. Although widely validated, generic measures may have a lower sensitivity to HF-specific symptom domains compared with disease-specific instruments, including the Kansas City Cardiomyopathy Questionnaire (KCCQ). In particular, domains highly relevant to HFpEF, including exertional intolerance, fatigue, and fluid-related symptoms may be insufficiently captured, potentially leading to underestimation of disease burden, especially in clinically stable patients. At the same time, the use of EQ-5D-5L offers several advantages. The instrument is simple, cost-free, and widely validated across different HF populations [10,46,47,48,49]. Moreover, given the high burden of comorbidities in HFpEF, which substantially influence overall health status beyond cardiac-specific symptoms, a generic health-related QoL instrument may provide a more comprehensive assessment of patient-reported outcomes. Importantly, EQ-5D-5L enables derivation of a standardized utility index suitable for statistical modeling, cross-population comparison, and health economic analysis [47].

In the present cohort, health-related QoL was characterized by substantial interindividual variability rather than uniformly severe impairment, indicating heterogeneous functional adaptation to a comparable clinical phenotype. The median EQ-5D index of 0.76 and median EQ-5D-5L VAS of 60% indicated moderate QoL impairment, which is aligned with previously reported data from randomized clinical trials [46]. Similar to other studies, domain-level analysis suggested that limitations were primarily driven by physical mobility and activities of daily living, whereas pain and psychological domains were less prominently affected. This pattern supports the interpretation that QoL impairment in HFpEF predominantly reflects functional restriction and comorbidity burden rather than isolated symptom intensity or emotional distress [46,47].

Decreased QoL correlated with older age, female sex, comorbidity burden and atrial fibrillation, degree of renal impairment, as well as with NYHA functional class and the presence of peripheral edema. However, the step 1 hierarchical model including demographic and comorbid characteristics explained only 20.4% of EQ-5D utility index variance. We note that NYHA class, despite significant association with QoL, was not included in the model, due to strong correlation with other variables (especially 6MWT-derived) and subjectivity in quantification.

Beyond cohort characterization, the main association observed in the analysis is the dominant role of functional limitation in shaping patient-reported health status. Although all three 6MWT-derived variables showed significant association with EQ-5D index, following multicollinearity rules, only a percentage of calculated distance was used, due to higher specificity (absolute distance) and objectivity (Borg scale). Adding a percentage of calculated distance in step 2 led to a nearly 2-fold relative increase in the explained QoL variance. The magnitude of the functional contribution was not only statistically robust but also clinically interpretable: stratification of patients by predicted QoL quartiles revealed a monotonic increase in the observed EQ-5D index values across successive quartiles once functional capacity was incorporated into the model, a pattern that was absent in the purely clinical model (Figure 1a,b). This observation aligns with prior studies demonstrating associations between functional capacity and patient-reported outcomes in HFpEF and other heart failure phenotypes [5,15,17,18,50]. Objective assessment of functional capacity integrates central hemodynamic reserve, chronotropic competence, ventricular–arterial coupling, peripheral oxygen utilization, skeletal muscle performance, and autonomic regulation, thereby reflecting the cumulative physiological constraints shaping daily activity tolerance and perceived well-being [13,14,15,16,51]. However, most previous analyses have examined functional performance, biomarkers, or QoL in isolation or within selected trial populations, limiting external validity and mechanistic integration. By applying a hierarchical modeling framework in a real-world cohort, the present analysis quantitatively demonstrates the incremental explanatory value of objective functional capacity beyond baseline clinical characteristics. Such findings underscore the central role of functional limitation as the primary driver of patient-reported health status in HFpEF, more closely aligned with daily functional experience than isolated clinical descriptors or laboratory markers [5,15,50].

Beyond functional performance, inflammatory activation emerged as an independent, albeit more modest, contributor to QoL impairment. IL-6 remained inversely associated with EQ-5D index values after adjustment for demographic factors, renal function, atrial fibrillation, and exercise capacity. In addition, adding IL-6 in step 3 of the hierarchical model resulted in a modest but statistically significant increase in explained variance. This finding is consistent with the paradigm of HFpEF as a systemic inflammatory disorder driven by comorbidity-related endothelial dysfunction and microvascular inflammation [19,20,21,22,23,24]. Chronic low-grade inflammation promotes myocardial stiffening, impaired nitric oxide signaling, and adverse ventricular–vascular coupling but also exerts extracardiac effects on skeletal muscle metabolism, mitochondrial efficiency, and peripheral oxygen utilization [19,20,24]. These mechanisms may contribute to fatigue, exertional intolerance, and reduced functional autonomy, thereby influencing quality of life independently of measured walking performance. Clinical studies have similarly linked elevated IL-6 and CRP levels with impaired exercise tolerance and adverse outcomes in HFpEF [25,26,27,28,29,30,31,32,52].

Although NT-proBNP was negatively associated with EQ-5D index in univariable analysis, it did not remain independently associated with QoL after adjustment for functional capacity and IL-6. In an exploratory hierarchical model in which biomarkers were entered before functional capacity, NT-proBNP showed a borderline association with QoL; however, this association was no longer present after the addition of 6MWT performance (Supplementary Table S3). This pattern should be interpreted only as evidence of overlapping explanatory information between NT-proBNP, functional capacity, and patient-reported health status, rather than as evidence of mediation or causality. No formal mediation analysis was performed. As per the literature, in HFpEF, natriuretic peptides retain diagnostic and prognostic relevance but may be influenced by comorbidities and extracardiac factors, which could partly explain the limited concordance between natriuretic peptide levels and patient-reported QoL observed in the published data [53,54,55]. Natriuretic peptides primarily reflect myocardial wall stress and filling pressures, which may be, theoretically, only weakly associated with subjective symptom burden and adaptive functional limitation in clinically stable HFpEF patients, particularly in the presence of obesity, multiple comorbidities, and early stages of the disease where hemodynamics are altered only in exertion [12,56,57,58].

Collectively, these findings support a conceptual framework in which QoL in HFpEF is determined predominantly by integrated functional limitation, with biological disease activity exerting a secondary but independent influence. From a clinical perspective, this emphasizes the importance of systematic functional assessment in the routine evaluation of HFpEF patients, beyond reliance on symptom class or biomarker levels alone [2,50]. At the same time, strategies aimed at modulating inflammatory pathways and comorbidity-driven systemic dysfunction may provide a complementary benefit, particularly in selected inflammatory phenotypes [50].

Several limitations should be acknowledged. The study was conducted in a single center with a moderate sample size, which may limit generalizability and statistical power for detecting smaller effects. The present study is cross-sectional in design and does not include a longitudinal assessment of treatment response or changes in functional or biomarker parameters over time. Therefore, causal inference and evaluation of intervention effects are limited. Quality of life was assessed using a generic instrument, which may be less sensitive to heart failure-specific symptom domains compared with disease-specific questionnaires (such as KCCQ). Echocardiographic parameters were not consistently available across the study cohort, with a substantial proportion of data missing from the registry, particularly for key indices such as E/e′ ratio and left atrial volume index (86/110 missing). Their inclusion in multivariable analyses would have substantially reduced the effective sample size and potentially introduced selection bias. No sensitivity or subgroup analyses were performed in patients with available echocardiographic data, as this subset was not considered representative of the overall cohort. This limitation should be acknowledged, as echocardiographic markers of diastolic dysfunction provide important complementary information regarding disease severity and hemodynamic status. In addition, the biomarker panel was restricted to selected inflammatory and cardiac markers and does not capture the full complexity of the neurohormonal, metabolic, and morpho-functional pathways involved in HFpEF. Although rigorous steps were taken to minimize multicollinearity and model instability, residual confounding cannot be fully excluded.

Future studies should extend these observations in larger, multi-center cohorts with longitudinal follow-up, integrating repeated functional assessments, broader biomarker profiling, and advanced imaging phenotyping.

## 5. Conclusions

In this real-world cohort of patients with HFpEF, objective functional capacity emerged as the principal determinant of health-related QoL, substantially outweighing the explanatory contribution of traditional clinical characteristics and cardiac biomarkers. Inflammatory activity, represented by IL-6, provided a modest but independent signal, whereas NT-proBNP did not retain an independent association after accounting for functional performance. These findings support a conceptual model in which patient-reported health status in HFpEF is more strongly associated with integrated functional limitation than with traditional clinical characteristics or NT-proBNP levels, while inflammatory activity provides a smaller independent explanatory contribution.

## Figures and Tables

**Table 1:** Baseline demographic, clinical, and laboratory characteristics of the cohort.

VARIABLE		Percentage (Number of Case) or Median (IQR)
SOCIO-DEMOGRAPHIC CHARACTERISTICS		
Sex	Male	47.3% (n = 52)
	Female	52.7% (n = 58)
Age [years]		72.0 (68.0–76.0)
COMORBIDITIES		
Arterial hypertension		94.5% (n = 104)
Hyperlipidemia		72.7% (n = 80)
Diabetes		45.5% (n = 50)
Atrial fibrillation		41.8% (n = 46)
Chronic kidney disease (eGFR < 60 mL/min/1.73 m^2^)		37.3% (n = 41)
Anemia		26.4% (n = 29)
Body mass index	<25 kg/m^2^	20.0% (n = 22)
	25.00–29.99 kg/m^2^	24.5% (n = 27)
	>30.00 kg/m^2^	55.5% (n = 61)
Charlson comorbidity index		4.0 (3.0–5.0)
CLINICAL CHARACTERISTICS		
Duration of symptoms to diagnosis [months]		12.0 (8.0–20.0)
Duration of symptoms to enrolment [months]		15.0 (9.0–21.2)
NYHA class	NYHA II	73.6% (n = 81)
	NYHA III	26.4% (n = 29)
Peripheral edema		80.0% (n = 88)
SELECTED LABORATORY MEASUREMENTS		
CRP [mg/L]		5.1 (1.7–6.8)
IL-6 [pg/mL]		5.4 (3.0–7.7)
NT-proBNP [pg/mL]		566.0 (272.0–1370.0)
eGFR [mL/min/1.73 m^2^]		65.5 (49.5–82.2)

**Table 2:** Functional capacity and health-related QoL assessment.

VARIABLE		Percentage (Number of Case) or Median (IQR)
6-MINUTE WALK TEST		
Distance covered [meters]		340.0 (280.0–392.2)
Percentage of calculated distance covered [percentage]		75.9 (62.7–83.8)
Borg scale		3.5 (3.0–5.0)
Borg scale categories	0–4	65.5% (n = 72)
	5–10	34.5% (n = 38)
QUALITY OF LIFE MEASUREMENTS		
EQ-5D utility index [index]		0.76 (0.67–0.88)
EQ-5D-5L VAS [percentage]		60.0 (50.0–61.2)
Mobility domain	“no problems”	15.5% (n = 17)
	“slight problems”	43.6% (n = 48)
	“moderate problems”	34.5% (n = 38)
	“severe problems”	6.4% (n = 7)
	“inability to walk”	0.0% (n = 0)

**Table 3:** Univariable determinants of health-related quality of life.

VARIABLE		EQ-5D-5L Index Median (IQR) or Spearman’s Coefficient	*p*
Sex	Male	0.80 (0.74–0.88)	0.002
	Female	0.73 (0.56–0.82)	
Age [years]		−0.245	0.001
Atrial fibrillation	Yes	0.73 (0.54–0.81)	0.008
	No	0.78 (0.70–0.89)	
BMI ≥ 30 kg/m^2^	Yes	0.75 (0.70–0.88)	0.766
	No	0.76 (0.67–0.87)	
CCI		−0.308	0.001
NYHA	II	0.81 (0.74–0.82)	<0.001
	III	0.53 (0.46–0.70)	
Peripheral edema	Yes	0.74 (0.65–0.82)	0.001
	No	0.87 (0.76–0.93)	
Percentage of calculated distance covered		0.496	<0.001
Borg scale		−0.596	<0.001
eGFR [mL/min/1.73 m^2^]		0.357	<0.001
CRP [mg/L]		−0.369	<0.001
IL-6 [pg/mL]		−0.441	<0.001
NT-proBNP [pg/mL]		−0.426	<0.001

**Table 4:** Multivariable hierarchical regression analysis of factors associated with health-related quality of life.

Independent Variable	Model 1	Model 2	Model 3	
	B	B	β	B (95% CI)
Step 1: demographics and comorbidities				
Age	−0.039	−0.060	−0.068	−0.002 (−0.006–0.002)
Female sex	−0.253 **	−0.282 ***	−0.283 ***	−0.097 (−0.146–−0.047)
Atrial fibrillation	0.237 **	0.114	0.103	0.036 (−0.020–0.091)
eGFR	0.271 **	0.263 **	0.243 **	0.002 (0.001–0.003)
R^2^	0.235	/		
Step 2: addition of functional capacity (6MWT)				
% of predicted 6MWT distance	/	0.483 ***	0.376 ***	+0.004 (0.002–0.006)
R^2^	/	0.452		
ΔR^2^	/	+0.217		
Step 3: addition of cardiac and inflammatory biomarkers				
NT-proBNP ^	/	/	−0.046	−0.006 (−0.032–+0.020)
IL-6 ^	/	/	−0.185 *	−0.046 (−0.087–−0.006)
R^2^	/	/	0.468	
ΔR^2^	/	/	+0.042	

ΔR^2^—change in R square; “*”—statistical significance at <0.05 level; “**”—statistical significance at <0.01 level; “***”—statistical significance at <0.001 level; “^”—computed values using the ln function.

## Data Availability

The dataset is available on request from the authors.

## Supplementary Materials

The following supporting information can be downloaded at: https://www.mdpi.com/article/10.3390/biomedicines14061270/s1, Table S1: Quality of life assessment by the EQ-5D-5L domains; Table S2: Sensitivity analysis for final regression model regarding comorbidities; Table S3: Hierarchical regression models with biomarkers entered prior to functional capacity.

## Abbreviations

The following abbreviations are used in this manuscript:

6MWT	Six-minute walk test
CCI	Charlson Comorbidity Index
CRP	C-reactive protein
GDMT	Guideline-directed medical therapy
eGFR	estimated Glomerular Filtration Rate
HFpEF	Heart Failure with preserved Ejection Fraction
HFrEF	Heart Failure with reduced Ejection Fraction
IL-6	Interleukin-6
IQR	Interquartile Range
NT-proBNP	N-terminal pro-B-type natriuretic peptide
NYHA	New York Heart Association
QoL	Quality of Life
VAS	Visual Analog Scale

## References

[1] Formiga F., Nuñez J., Castillo Moraga M.J., Cobo Marcos M., Egocheaga M.I., García-Prieto C.F., Trueba-Sáiz A., Matalí Gilarranz A., Fernández Rodriguez J.M. (2024). Diagnosis of heart failure with preserved ejection fraction: A systematic narrative review of the evidence. Heart Fail. Rev..

[2] Shah S.J., Borlaug B.A., Kitzman D.W., McCulloch A.D., Blaxall B.C., Agarwal R., Chirinos J.A., Collins S., Deo R.C., Gladwin M.T. (2020). Research priorities for heart failure with preserved ejection fraction: National Heart, Lung, and Blood Institute Working Group summary. Circulation.

[3] Campbell P., Rutten F.H., Lee M.M., Hawkins N.M., Petrie M.C. (2024). Heart failure with preserved ejection fraction: Everything the clinician needs to know. Lancet.

[4] Kapelios C.J., Shahim B., Lund L.H., Savarese G. (2023). Epidemiology, clinical characteristics and cause-specific outcomes in heart failure with preserved ejection fraction. Card. Fail. Rev..

[5] Reddy Y.N.V., Rikhi A., Obokata M., Shah S.J., Lewis G.D., AbouEzzedine O.F., Dunlay S., McNulty S., Chakraborty H., Stevenson L.W. (2020). Quality of life in heart failure with preserved ejection fraction: Importance of obesity, functional capacity, and physical inactivity. Eur. J. Heart Fail..

[6] Ferreira J.P., Shah A.M., Claggett B.L., Pitt B., Lewis E.F., Solomon S.D., Zannad F. (2022). Cardiac structure and function and quality of life associations in HFpEF: An analysis from TOPCAT-Americas. Int. J. Cardiol..

[7] Kato N., Kinugawa K., Seki S., Shiga T., Hatano M., Yao A., Hirata Y., Kazuma K., Nagai R. (2011). Quality of life as an independent predictor for cardiac events and death in patients with heart failure. Circ. J..

[8] Kao G., Xu G., Zhang Y., Li C., Xiao J. (2024). Predictive value of quality of life as measured by KCCQ in heart failure patients: A meta-analysis. Eur. J. Clin. Investig..

[9] Xu J., Sun Y., Gong D., Fan Y. (2023). Association between disease-specific health-related quality of life and all-cause mortality in patients with heart failure: A meta-analysis. Curr. Probl. Cardiol..

[10] Sepehrvand N., Savu A., Spertus J.A., Dyck J.R.B., Anderson T., Howlett J., Paterson I., Oudit G.Y., Kaul P., McAlister F.A. (2020). Change of health-related quality of life over time and its association with patient outcomes in patients with heart failure. J. Am. Heart Assoc..

[11] Raphael C., Briscoe C., Davies J., Whinnett Z.I., Manisty C., Sutton R., Mayet J., Francis D.P. (2007). Limitations of the New York Heart Association functional classification system and self-reported walking distances in chronic heart failure. Heart.

[12] Pfeffer M.A., Shah A.M., Borlaug B.A. (2019). Heart failure with preserved ejection fraction in perspective. Circ. Res..

[13] Stoicescu L., Crişan D., Morgovan C., Avram L., Ghibu S. (2024). Heart failure with preserved ejection fraction: The pathophysiological mechanisms behind the clinical phenotypes and the therapeutic approach. Int. J. Mol. Sci..

[14] Fraser A.G. (2018). What limits functional capacity in heart failure with preserved ejection fraction? Unravelling the knots of an enigma. JACC Heart Fail..

[15] Scrutinio D., Guida P., Passantino A. (2024). Functional limitation predicts mortality in heart failure with preserved ejection fraction. Eur. J. Intern. Med..

[16] Borlaug B.A. (2014). The pathophysiology of heart failure with preserved ejection fraction. Nat. Rev. Cardiol..

[17] Cavero-Redondo I., Saz-Lara A., Bizzozero-Peroni B., Núñez-Martínez L., Díaz-Goñi V., Calero-Paniagua I., Matínez-García I., Pascual-Morena C. (2024). Accuracy of the 6-minute walk test for assessing functional capacity in patients with heart failure with preserved ejection fraction and other chronic cardiac pathologies. Sports Med. Open.

[18] Pepera G., Antoniou V., Karagianni E., Batalik L., Su J.J. (2025). Validity and reliability of the six-minute walking test compared to cardiopulmonary exercise test in individuals with heart failure: Systematic review and meta-analysis. J. Clin. Med..

[19] Mesquita T., Lin Y.N., Ibrahim A. (2021). Chronic low-grade inflammation in heart failure with preserved ejection fraction. Aging Cell.

[20] Paulus W.J., Tschöpe C. (2013). A novel paradigm for heart failure with preserved ejection fraction: Comorbidities drive myocardial dysfunction and remodeling through coronary microvascular endothelial inflammation. J. Am. Coll. Cardiol..

[21] Sanders-van Wijk S., Tromp J., Beussink-Nelson L., Hage C., Svedlund S., Saraste A., Swat S.A., Sanchez C., Njoroge J., Tan R.S. (2020). Proteomic evaluation of the comorbidity-inflammation paradigm in heart failure with preserved ejection fraction. Circulation.

[22] Van Linthout S., Tschöpe C. (2017). Inflammation—Cause or consequence of heart failure or both?. Curr. Heart Fail. Rep..

[23] Cornuault L., Rouault P., Duplàa C., Couffinhal T., Renault M.A. (2022). Endothelial dysfunction in heart failure with preserved ejection fraction: What are the experimental proofs?. Front. Physiol..

[24] Murphy S.P., Kakkar R., McCarthy C.P., Januzzi J.L. (2020). Inflammation in heart failure: JACC state-of-the-art review. J. Am. Coll. Cardiol..

[25] Alogna A., Koepp K.E., Sabbah M., Espindola Netto J.M., Jensen M.D., Kirkland J.L., Lam C.S.P., Obokata M., Petrie M.C., Ridker P.M. (2023). Interleukin-6 in patients with heart failure and preserved ejection fraction. JACC Heart Fail..

[26] Berger M., März W., Niessner A., Delgado G., Kleber M., Scharnagl H., Marx N., Schuett K. (2024). IL-6 and hsCRP predict cardiovascular mortality in patients with heart failure with preserved ejection fraction. ESC Heart Fail..

[27] Markousis-Mavrogenis G., Tromp J., Ouwerkerk W., Devalaraja M., Anker S.D., Cleland J.G., Dickstein K., Filippatos G.S., van der Harst P., Lang C.C. (2019). The clinical significance of interleukin-6 in heart failure: Results from the BIOSTAT-CHF study. Eur. J. Heart Fail..

[28] Koller L., Kleber M., Goliasch G., Sulzgruber P., Scharnagl H., Silbernagel G., Grammer T., Delgado G., Tomaschitz A., Pilz S. (2014). C-reactive protein predicts mortality in patients referred for coronary angiography and symptoms of heart failure with preserved ejection fraction. Eur. J. Heart Fail..

[29] Mooney L., Jackson C.E., Adamson C., McConnachie A., Welsh P., Myles R.C., McMurray J.J.V., Jhund P.S., Petrie M.C., Lang N.N. (2023). Adverse outcomes associated with interleukin-6 in patients recently hospitalized for heart failure with preserved ejection fraction. Circ. Heart Fail..

[30] Albar Z., Albakri M., Hajjari J., Karnib M., Janus S.E., Al-Kindi S.G. (2022). Inflammatory markers and risk of heart failure with reduced to preserved ejection fraction. Am. J. Cardiol..

[31] Chia Y.C., Kieneker L.M., van Hassel G., Binnenmars S.H., Nolte I.M., van Zanden J.J., van der Meer P., Navis G., Voors A.A., Bakker S.J.L. (2021). Interleukin-6 and development of heart failure with preserved ejection fraction in the general population. J. Am. Heart Assoc..

[32] Pugliese N.R., Pellicori P., Filidei F., De Biase N., Maffia P., Guzik T.J., Masi S., Taddei S., Cleland J.G.F. (2023). Inflammatory pathways in heart failure with preserved left ventricular ejection fraction: Implications for future interventions. Cardiovasc. Res..

[33] McDonagh T.A., Metra M., Adamo M., Gardner R.S., Baumbach A., Böhm M., Burri H., Butler J., Čelutkienė J., Chioncel O. (2024). 2023 focused update of the 2021 ESC guidelines for the diagnosis and treatment of acute and chronic heart failure. Eur. J. Heart Fail..

[34] ATS Committee on Proficiency Standards for Clinical Pulmonary Function Laboratories (2002). ATS statement: Guidelines for the six-minute walk test. Am. J. Respir. Crit. Care Med..

[35] Johnson M.J., Close L., Gillon S.C., Molassiotis A., Lee P.H., Farquhar M.C. (2016). Use of the modified Borg scale and numerical rating scale to measure chronic breathlessness: A pooled data analysis. Eur. Respir. J..

[36] Devlin N.J., Shah K.K., Feng Y., Mulhern B., van Hout B. (2018). Valuing health-related quality of life: An EQ-5D-5L value set for England. Health Econ..

[37] Green S.B. (1991). How Many Subjects Does It Take To Do A Regression Analysis. Multivar. Behav. Res..

[38] Solomon S.D., McMurray J.J.V., Anand I.S., Ge J., Lam C.S.P., Maggioni A.P., Martinez F., Packer M., Pfeffer M.A., Pieske B. (2019). Angiotensin-neprilysin inhibition in heart failure with preserved ejection fraction. N. Engl. J. Med..

[39] Pitt B., Pfeffer M.A., Assmann S.F., Boineau R., Anand I.S., Claggett B., Clausell N., Desai A.S., Diaz R., Fleg J.L. (2014). Spironolactone for heart failure with preserved ejection fraction. N. Engl. J. Med..

[40] Shah A.M., Solomon S.D. (2012). Phenotypic and pathophysiological heterogeneity in heart failure with preserved ejection fraction. Eur. Heart J..

[41] Kapłon-Cieślicka A., Lund L.H. (2021). Do we need a definition of acute heart failure with preserved ejection fraction?. Ann. Med..

[42] Kapłon-Cieślicka A., Laroche C., Crespo-Leiro M.G., Coats A.J.S., Anker S.D., Filippatos G., Maggioni A.P., Hage C., Lara-Padrón A., Fucili A. (2020). Is heart failure misdiagnosed in hospitalized patients with preserved ejection fraction?. ESC Heart Fail..

[43] Palazzuoli A., Caravita S., Paolillo S., Ghio S., Tocchetti C.G., Ruocco G., Correale M., Ambrosio G., Perrone Filardi P., Senni M. (2021). Current gaps in HFpEF trials: Time to reconsider patients’ selection and to target phenotypes. Prog. Cardiovasc. Dis..

[44] Pieske B., Tschöpe C., de Boer R.A., Fraser A.G., Anker S.D., Donal E., Edelmann F., Fu M., Guazzi M., Lam C.S.P. (2019). How to diagnose heart failure with preserved ejection fraction: The HFA–PEFF diagnostic algorithm: A consensus recommendation from the Heart Failure Association (HFA) of the European Society of Cardiology (ESC). Eur. Heart J..

[45] Migas S., Ellis M.L., Wrona B., Rivero Sanz E., Brownrigg J., Strauss O., Ahmed F.Z. (2024). Missed opportunities in heart failure diagnosis and management: Study of an urban UK population. ESC Heart Fail..

[46] Seo M., Watanabe T., Yamada T., Yano M., Hayashi T., Nakagawa A., Nakagawa Y., Tamaki S., Yasumura Y., Sotomi Y. (2023). The clinical relevance of quality of life in heart failure patients with preserved ejection fraction. ESC Heart Fail..

[47] Yang M., Kondo T., Talebi A., Jhund P.S., Docherty K.F., Claggett B.L., Vaduganathan M., Bachus E., Hernandez A.F., Lam C.S.P. (2025). EuroQol 5-dimension questionnaire in heart failure with reduced, mildly reduced, and preserved ejection fraction: A patient-level analysis of DAPA-HF and DELIVER. JACC Heart Fail..

[48] Thomas M., Jones P.G., Cohen D.J., Suzanne A.V., Magnuson E.A., Wang K., Thourani V.H., Fonarow G.C., Sandhu A.T., Spertus J.A. (2021). Predicting the EQ-5D utilities from the Kansas City Cardiomyopathy Questionnaire in patients with heart failure. Eur. Heart J. Qual. Care Clin. Outcomes.

[49] Berg J., Lindgren P., Mejhert M., Edner M., Dahlström U., Kahan T. (2015). Determinants of Utility Based on the EuroQol Five-Dimensional Questionnaire in Patients with Chronic Heart Failure and Their Change Over Time: Results from the Swedish Heart Failure Registry. Value Health.

[50] Pandey A., Khera R., Park B., Haykowsky M., Borlaug B.A., Lewis G.D., Kitzman D.W., Butler J., Berry J.D. (2018). Relative impairments in hemodynamic exercise reserve parameters in heart failure with preserved ejection fraction: A study-level pooled analysis. JACC Heart Fail..

[51] D’Amario D., Migliaro S., Borovac J.A., Restivo A., Vergallo R., Galli M., Leone A.M., Montone R.A., Niccoli G., Aspromonte N. (2019). Microvascular dysfunction in heart failure with preserved ejection fraction. Front. Physiol..

[52] Kalogeropoulos A., Georgiopoulou V., Psaty B.M., Rodondi N., Smith A.L., Harrison D.G., Liu Y., Hoffmann U., Bauer D.C., Newman A.B. (2010). Inflammatory markers and incident heart failure risk in older adults: The Health ABC study. J. Am. Coll. Cardiol..

[53] Meijers W.C., Hoekstra T., Jaarsma T., van Veldhuisen D.J., de Boer R.A. (2016). Patients with heart failure with preserved ejection fraction and low levels of natriuretic peptides. Neth. Heart J..

[54] Kristensen S.L., Mogensen U.M., Jhund P.S., Rørth R., Anand I.S., Carson P.E., Desai A.S., Pitt B., Pfeffer M.A., Solomon S.D. (2019). N-terminal pro-B-type natriuretic peptide levels for risk prediction in patients with heart failure and preserved ejection fraction according to atrial fibrillation status. Circ. Heart Fail..

[55] Li J., Lei L., Wang W., Li Y., Yu Y., Pu B., Peng Y., Huo X., Zhang L. (2025). Patient-reported health status vs. N-terminal pro-B-type natriuretic peptide levels in patients with acute heart failure. Chin. Med. J..

[56] Teramoto K., Nochioka K., Sakata Y., Nishimura K., Shimokawa H., Yasuda S. (2025). Heart failure with preserved ejection fraction and lower natriuretic peptide: Clinical characteristics and change in natriuretic peptide levels. J. Am. Heart Assoc..

[57] Bayes-Genis A., Cediel G., Domingo M., Codina P., Santiago E., Lupón J. (2022). Biomarkers in heart failure with preserved ejection fraction. Card. Fail. Rev..

[58] Tanase D.M., Radu S., Al Shurbaji S., Baroi G.L., Florida Costea C., Turliuc M.D., Ouatu A., Floria M. (2019). Natriuretic peptides in heart failure with preserved left ventricular ejection fraction: From molecular evidences to clinical implications. Int. J. Mol. Sci..

