# Supplementary material for: Relative Contributions of Functional Capacity and Inflammatory Activity to Quality of Life in Heart Failure with Preserved Ejection Fraction
Source: Biomedicines. 2026 Jun 2;14(6):1270. doi: 10.3390/biomedicines14061270 (PMC13297599; doi:10.3390/biomedicines14061270)
Supplement: Supplementary file 1 [file biomedicines-14-01270-s001.zip › biomedicines-4205512-supplementary.pdf]

Supplementary Table S1. Quality of life assessment by the EQ-5D-5L domains

EQ-5D-5L domain	Likert scale	Percentage (number of cases)
Mobility	"no problems"	15.5% (n=17)
	"slight problems"	43.6% (n=48)
	"moderate problems"	34.5% (n=38)
	"severe problems"	6.4% (n=7)
	"inability to walk"	0.0% (n=0)
Self-care	"no problems"	66.% (n= 73)
	"slight problems"	25.5% (n= 28)
	"moderate problems"	6.4% (n= 7)
	"severe problems"	1.8% (n= 2)
	"inability to wash/dress myself"	0.0% (n= 0)
Usual activities	"no problems"	3.6% (n= 4)
	"slight problems"	31.8% (n= 35)
	"moderate problems"	57.3% (n= 63)
	"severe problems"	7.3% (n= 8)
	"inability to perform daily activities"	0.0% (n= 0)
Pain/discomfort	"no pain/discomfort"	60.9% (n= 67)
	"slight pain/discomfort"	34.5% (n= 38)
	"moderate pain/discomfort"	4.5% (n= 5)
	"severe pain/discomfort"	0.0% (n= 0)
	"extreme pain/discomfort"	0.0% (n= 0)
Anxiety/depression	"not anxious/depressed"	35.5% (n= 39)
	"slightly anxious/depressed"	35.5% (n= 39)
	"moderately anxious/depressed"	13.6% (n= 15)
	"severely anxious/depressed"	15.5% (n= 17)
	"extremely anxious/depressed"	0.0% (n= 0)

Supplementary Table S2. Sensitivity analysis for final regression model regarding comorbidities

	Original Model	Sensitivity 1	Sensitivity 2	Sensitivity 3	Sensitivity 4
**Additional variable**	/	Diabetes	Hypertension	BMI ≥ 30 kg/m²	CCI
**β for 6MWT%**	0.376	0.343	0.355	0.341	0.330
**P for 6MWT%**	< 0.001	< 0.001	< 0.001	< 0.001	0.001
**β for IL-6 ^**	-0.185	-0.209	-0.184	-0.205	-0.205
**P for IL-6 ^**	0.018	0.012	0.033	0.018	0.016
**β for NT-proBNP ^**	-0.046	-0.058	-0.036	-0.045	-0.049
**P for NT-proBNP ^**	0.563	0.524	0.703	0.649	0.599
**β for added variable**	/	0.167	-0.081	0.022	-0.060
**P for added variable**	/	0.114	0.297	0.786	0.570
**R^2**	0.468	0.480	0.468	0.463	0.464

Supplementary Table S2 legend: "^" – log-transformed values using the natural logarithm; 6MWT% - Percentage of predicted six minute walk test distance; BMI – Body Mass Index; CCI – Charlson Comorbidity Index; IL-6 – Interleukin 6; NT-proBNP - N-terminal pro-B-type natriuretic peptide.

Supplementary Table S3. Hierarchical regression models with biomarkers entered prior to functional capacity.

Independent variable	Model 1	Model 2	Model 3	
	B	β	β	
**Step 1: demographics and comorbidities**				
Age	-0.039	-0.060	-0.068	-0.002 (-0.006 - 0.002)
Female sex	-0.253**	-0.282***	-0.283***	-0.097 (-0.146 - -0.047)
Atrial fibrillation	0.237**	0.114	0.103	0.036 (-0.020 - 0.091)
eGFR	0.271**	0.263**	0.243**	0.002 (0.001 - 0.003)
R^2	0.235	/		
**Step 2: addition of cardiac and inflammatory biomarkers**				
NT-proBNP ^	/	-0.185*	-0.045	-0.006 (-0.032 - +0.020)
IL-6 ^	/	-0.317***	-0.185*	-0.046 (-0.087 - -0.006)
R^2	/	0.392		
ΔR^2	/	0.157		
**Step 3: addition of functional capacity (6MWT)**				
% of predicted 6MWT distance	/	/	0.376***	0.004 (0.002 - 0.006)
R^2	/	/	0.468	
ΔR^2	/	/	0.076	

Supplementary Table S3 legend: ΔR2 - change in R square; "*" - statistical significance at < 0.05 level; "**" - statistical significance at < 0.01 level; "***" - statistical significance at < 0.001 level; "^" – log-transformed values using the natural logarithm.
